# Survival of RNA Replicators Is Much Easier in Protocells Than in Surface-Based, Spatial Systems

**DOI:** 10.3390/life9030065

**Published:** 2019-08-07

**Authors:** Vismay Shah, Jonathan de Bouter, Quinn Pauli, Andrew S. Tupper, Paul G. Higgs

**Affiliations:** 1 Origins Institute and Department of Physics and Astronomy, McMaster University, Hamilton, ON L8S 4L8, Canada; 2 Origins Institute and Department of Biochemistry and Biomedical Sciences, McMaster University, Hamilton, ON L8S 4L8, Canada

**Keywords:** RNA World, polymerase, error threshold, protocell, membranes, spatial lattice model, evolution of cooperation, parasites

## Abstract

In RNA-World scenarios for the origin of life, replication is catalyzed by polymerase ribozymes. Replicating RNA systems are subject to invasion by non-functional parasitic strands. It is well-known that there are two ways to avoid the destruction of the system by parasites: spatial clustering in models with limited diffusion, or group selection in protocells. Here, we compare computational models of replication in spatial models and protocells as closely as possible in order to determine the relative importance of these mechanisms in the RNA World. For the survival of the polymerases, the replication rate must be greater than a minimum threshold value, *k_min_*, and the mutation rate in replication must be less than a maximum value, *M_max_*, which is known as the error threshold. For the protocell models, we find that *k_min_* is substantially lower and *M_max_* is substantially higher than for the equivalent spatial models; thus, the survival of polymerases is much easier in protocells than on surfaces. The results depend on the maximum number of strands permitted in one protocell or one lattice site in the spatial model, and on whether replication is limited by the supply of monomers or the population size of protocells. The substantial advantages that are seen in the protocell models relative to the spatial models are robust to changing these details. Thus, cooperative polymerases with limited accuracy would have found it much easier to operate inside lipid compartments, and this suggests that protocells may have been a very early step in the development of life. We consider cases where parasites have an equal replication rate to polymerases, and cases where parasites multiply twice as fast as polymerases. The advantage of protocell models over spatial models is increased when the parasites multiply faster.

## 1. Introduction

The most widely studied theory for the origin of life—specifically, the transition from a mixture of prebiotic chemical components to a living system that is sustained by autocatalytic replication—is the RNA World theory [1,2,3,4,5,6,7]. Most versions of the RNA World propose the existence of RNA polymerase ribozymes that use a second strand as a template to synthesize the complementary strand to the template. RNA polymerase ribozymes have been experimentally developed while using in vitro evolution with maximum template lengths of 200 nucleotides and per-base error rates of a few percent [8,9,10]. Although none of the laboratory ribozymes is yet able to replicate its own sequence, these experiments point to the likelihood that ribozymes working in this way could have supported life in its earliest stages.

Replicating systems containing polymerase ribozymes are subject to invasion by non-functional sequences that act as parasites. Parasite strands are likely to frequently arise, either as a result of chemical synthesis of new random sequences, or as a result of errors in the replication of functional strands. It is known that parasites destroy the replicating system unless there is a mechanism that promotes cooperation among groups of functional polymerases. Two such mechanisms have been widely studied: spatial clustering of polymerases in two-dimensional lattice models representing RNA strands bound to a surface [11,12,13,14,15,16,17,18], and group selection of polymerases inside protocell compartments [19,20,21]. Spatial clustering is beneficial to polymerases, because a neighbour of a polymerase is more likely to be another polymerase and less likely to be a parasite than it would be if sequences were randomly mixed. Protocell compartments benefit polymerases, because compartments with more functional strands and fewer parasites grow and divide more frequently. There have also been several experimental studies of replication inside artificial protocells [22,23,24,25]. It is unknown whether the first replicating molecules arose outside of compartments, and they were later encapsulated in membranes, or whether they arose inside a pre-existing system of simple membrane compartments. The ubiquity of cellular life today and the absence of current surface-based, non-encapsulated life forms suggest that cells arose rather early in the history of life. 

For polymerase ribozymes to survive, they must replicate faster than the rate at which they are destroyed by hydrolysis; hence, there is a minimum value of the replication rate constant, *k_min_,* which is required for survival. The polymerases must also replicate accurately enough to pass on their own sequence and avoid invasion by parasitic mutant sequences; hence, there is a maximum value of the mutation probability, *M_max_*, required for survival. RNA replicating systems are most likely to survive in systems with low *k_min_*, and high *M_max_*. Here, we study several alternative versions of protocell and spatial models for RNA replication in order to compare the values of *k_min_* and *M_max_*.

The maximum tolerable error rate in models of sequence replication is usually called the error threshold. The original error threshold theory of Eigen et al. [26] dealt with first order replication, meaning that one strand has the ability to make a second. This is applicable if the catalyst that replicates the strand is not part of the evolutionary model, as is the case in experiments where the RNA sequence evolve in the presence of a supply of Qβ replicase protein that is provided by the experimenter. Here, we deal with second-order catalysis, meaning that two molecules have the ability to make a third. This is applicable in the RNA World, if a polymerase ribozyme acts on a template strand to make a third strand. In both the first- and second-order replication problem, selection favors functional molecules and mutation creates non-functional molecules (or slower replicators with reduced functionality). In the first-order problem, selection arises due to the functional molecule, or master sequence, replicates faster than the mutant sequences. There is no need for spatial structure or protocells in the first-order model, because the master sequence survives by selective advantage even in the well-mixed case. On the other hand, in the second-order problem that was studied here, mutant sequences are parasites that cannot replicate themselves, and we assume that the rate at which parasites are copied by the polymerase is the same as the rate at which polymerases and complements are copied. There is no selective advantage of the polymerase in this case, and mutation favors the parasites. Therefore, the polymerases are destroyed by the parasites for any non-zero value of the mutation rate if the system is well-mixed. In the second-order case, either spatial structure or protocells are essential for the survival of the polymerases, as we have considered in several of our previous papers [6,16,17,18], because polymerases then have a selective advantage arising from clustering or group selection.

The simplest kind of deleterious mutation is one that destroys the function of the polymerase completely, without changing its ability to act as a template. We presume that such deleterious mutations will be frequent, because (i) a substantial fraction of point mutations in RNA sequences disrupt the secondary structure and (ii) a useful polymerase in the RNA World needs to be very insensitive to the sequence of the template, otherwise it cannot support further evolution and it cannot provide a means of replicating genes with other functions (for example, nucleotide synthetases [17]). Here, we focus on the case where the replication rate of the parasites is equal to the functional sequence, however the case of parasites whose replication rate is faster than that of the polymerases is also relevant, and it is considered at the end of this paper. In some cases, the advantage to the polymerase arising from clustering or group selection is sufficient to outweigh a large replication-rate advantage of the parasite (we consider a two-fold replication rate advantage in Section 3.4). One reason why parasites might replicate faster is if the mutation destroys the secondary structure of the polymerase, and the mutant sequence spends a larger fraction of its time in an unfolded state that is accessible as the template for another polymerase. A second reason might be that parasites could be shorter in sequence, and hence more rapidly replicated. We previously considered the case where short parasites are created by incomplete replication that terminates before the end of the strand is reached [18]. 

Although catalytic replication in the RNA World is second-order, first-order replication could also be relevant if it is non-enzymatic. We refer to first- and second-order rates as *r* and *k* respectively. We have previously considered cases where both *r* and *k* are considered in the model [16,27]. These earlier papers focus on the transition to life/replication, rather than the maintenance of replication in system that is already living, as we do here. Some small *r* rate is necessary for creating the first catalysts, but the *k* rate is likely to be much higher than the *r* rate once well-adapted polymerases are present; hence, we assume that *r* can be neglected. The relative rates of *r* and *k* are important when we consider the question of which kind of ribozymes came first [28]. If *r* was small, the first biological catalysts must have been polymerases, whereas if *r* was high (because non-enzymatic replication was intrinsically fast), then other kinds of catalysts that contribute to the synthesis of RNA and its precursors could have preceded polymerases. These scenarios are qualitatively different, as we discuss in [28]. Functional ribozymes are likely to be quite long (maybe 100 nucleotides or more). We do not know whether the replication of sequences of this length could be non-enzymatically possible; however, non-enzymatic replication of short oligomers seems likely, and it may have preceded the origin of ribozymes. We have referred to non-enzymatic replication of oligomers as “chemical evolution” [29]. Such a system would really be evolving, because sequence information in the oligomers would be passed on during replication. However, it would be distinct from the usual view of molecular evolution, which we have called “biological evolution”, because sequences would be selected based on physicochemical properties rather than encoded function, and because oligomers would be short enough to be synthesized from scratch as well as by replication of existing oligomers. In the chemical evolution case, diversity can arise by chemical synthesis as well as by mutations of existing sequences, whereas, in biological evolution, diversity arises only by mutation, because functional gene sequences (including well-adapted ribozymes in the RNA world) would be too long to be synthesized by means other than copying an existing template. We have argued that chemical evolution is a significant step on the path to life [29], and that, if evolution is considered to be a defining feature of life, then it is the presence of biological evolution that defines life, not simply chemical evolution.

We now return the principle question in the present paper. Assuming that replication in the RNA World is maintained by second-order polymerases, and that first-order non-enzymatic replication can be neglected at this stage, then how can we quantitatively compare spatial clustering and encapsulation in protocells as mechanisms allowing for the survival of polymerases? This requires us to think carefully about what the spatial models actually represent. In spatial lattice models of RNA replicators [11,12,13,14,15,16,17,18], it is usually assumed that only one strand is allowed per lattice site, and that a polymerase on one site replicates a template on a neighboring site. One way to view this would be to say that the lattice represents a two-dimensional surface, on which the strands are fixed. There is evidence that synthesis of short RNA oligomers can occur in the presence of clay surfaces [30] and in alkaline hydrothermal vent systems [31], but it is less clear whether minerals help in ribozyme function. It has been shown that clays can increase the rate of self-cleavage of the hammerhead ribozyme [32], but cleavage is not equivalent to replication. In a study of in vitro RNA evolution with and without the presence of clay [33], it was concluded that the effect of the clay was minimal, neither improving nor preventing the ability of RNA to evolve functional structures. Furthermore, ligases and polymerases are the most relevant laboratory ribozymes for replication in the RNA world [8,9,10], and these are not associated with mineral surfaces.

We suggest that the spatial models of replication are best viewed as representing the effects of confined geometry, slow diffusion, and spatial clustering of cooperating molecules, rather than literally representing molecules that are “stuck” on a surface. Spatial models require slow diffusion of molecules, so that clustering of polymerases arises. Nevertheless, some degree of motion is required so that replicating molecules can spread and encounters occur between polymerases and templates. One conceptual problem is that, if a molecule is stuck to a surface, it is difficult to see how it could slide along the surface without detaching. If it detached from the surface, then it would often diffuse away from the surface and be lost in open water. On the other hand, if the spatial lattice represents a confined geometry, such as pores in a rock [34], a mineral matrix [13], or cavities in which strands are trapped by thermophoresis [35], then diffusion will slowly occur and the molecules will remain within the restricted space.

In this paper, we consider the spatial models in which diffusion occurs slowly in a restricted space. When we think in this way, there is a small length scale (pore or cavity size) within which strands can quickly mix and interact, but motion of strands on large scales is controlled by slow diffusion. We can think of one lattice site in the models as a pore size, and the volume of this pore will control the maximum number of strands that can be on one site, which we call *S*_0_. One example of a model that is defined in this manner is featured in the study of Branciamore et al. [13]. In their paper, several types of autocatalytic replicators, each catalyzing one reaction in a metabolic network, could be present in a pore. Each pore was assigned a fitness that corresponded to the diversity of replicators it contained, with the requirement that at least one strand of each replicator type be present. Parasitic replicators were introduced through invasion and were also autocatalytic, competing with members of the network for resources without catalyzing any of the reactions [13]. In contrast, instead, we focus on a trans-acting polymerase. The parasitic sequences that we consider are fundamentally different: they cannot replicate without a polymerase present in the site. The polymerases may erroneously produce parasites from an improper replication. Hence, in our study, the central question is the maximum mutation rate the system can sustain rather than the number of different replicator species that can be sustained.

The minimum number of strands that must be allowed on a site if we want to allow for second order replication steps to occur on one site is three. The “two’s company and three’s a crowd” scheme that we previously studied [16,27], allows up to three strands per site for this reason. In the current paper, our object is to compare lattice models with protocell models; therefore, we want the underlying rules of the two types of models to be as similar as possible. In protocell models [19,20,21], it is typically assumed that the strands inside one protocell are well mixed and interact freely, but there is no interaction between strands in different protocells. Here, we want to make a direct analogy between one lattice site in the spatial models and one protocell in the protocell models. We define *S*_0_ in the protocell models as the number of strands at which cells divide. Thus, *S*_0_ controls the maximum number of strands per cell/site in both types of model. The rules for replication of strands are identical in the two types of models when we define the models in this way. This enables us to focus on the *differences* between spatial models and protocells: in spatial models, replication occurs locally on one lattice site and the diffusion of strands occurs between neighbouring sites, whereas in protocell models, replication occurs locally in one protocell, new cells arise when cells divide, there is no diffusion of strands between cells, and there is no spatial structure of the cells. 

An alternative way to compare spatial models and compartments is to use the Cellular Potts Model (CPM) [14], in which each compartment occupies multiple sites and only one strand is allowed per site. However, this model does not separate the effects of the compartments from the effects of spatial structure, because the compartments in the CPM are themselves on a lattice and they have spatial neighbours. Diffusion of strands can occur between neighboring compartments in the CPM, whereas in the protocell models that we use here, there is no diffusion between cells, simply growth, division, and death of cells. In some ways, the CPM model is closer to a spatial model in a restricted geometry than it is to a model of independent protocells. The study using the CPM [14] deliberately avoided making quantitative comparisons between the models with and without compartments, and concluded that it was impracticable to make a fair quantitative comparison. However, here, we have defined the rules of the models so that a fair quantitative comparison can be made, and such that there is either slow diffusion and spatial clustering or group selection in compartments, and not both. We will show that the outcome of this comparison is that there is a substantial quantitative advantage to the protocell models over the equivalent spatial models, both in terms of the minimum catalytic rate that is required for survival and the maximum error rate that can be tolerated.

## 2. Materials and Methods 

### 2.1. Overview of Models 

In this study, we compare protocell models and spatial lattice models in such a way that there is close analogy between one site in the lattice models and one protocell in the protocell models. Each lattice site (or each protocell) can hold multiple strands. Reactions that create and destroy strands occur locally on one site (or in one protocell) and are equivalently defined in the two kinds of models. Differences between the models are related to the dynamics of strands between sites (or protocells) and the factors that limit the replication of strands. 

Each strand is one of three types: a polymerase (P), a complementary sequence to the polymerase (C), or a non-functional strand, which we refer to as a parasite (X). Accurate replication of a P produces a C, and vice versa. If a point mutation occurs during replication of a P or C, an X is produced. The replication of an X always produces another X. We do not allow back mutations that produce P or C strands from replicating an X. Replication only occurs via the action of a polymerase catalyst, and we ignore non-enzymatic template-directed replication. There is a rate of breakdown of strands back to monomers that is assumed to be equal for the three types of strands.

**Figure 1:**
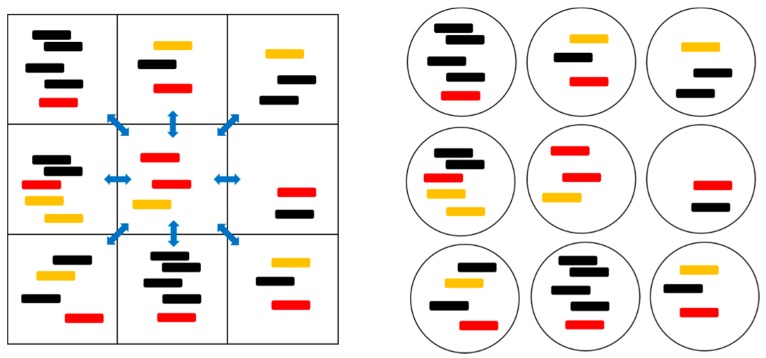
A cartoon representation of the spatial model with local diffusion dynamics (left) and the protocell models (right). The red strands are polymerases (P), orange strands are complements to polymerases (C), and black strands are parasites (X). The blue arrows indicate the possibility of diffusion to and from the eight neighboring sites.

Figure 1 and Table 1 summarize the models that were studied in this paper. In the two protocell models, division occurs when the number of strands in a cell, *S*, reaches a specified value, *S*_0_. This produces two daughter cells with the strands that were randomly divided between them. The number of protocells in the population, *N*, is a fixed parameter in the PCP model (Protocells with Constant Population), and is variable in the PML model (Protocells-Monomer Limited). In the PCP model, whenever a cell divides, another random cell is removed from the population to keep *N* fixed. This represents a situation where resources, such as lipids or available space, limit population growth. It is analogous to the standard Moran model that was used in population genetics [36]. In each model, there is a limiting factor *F* in the replication rates, which is required for preventing indefinite increase of either the population or the number of strands (details below in Section 2.2). In the PCP model, the population is already limited by fixing *N*, therefore no additional limiting factor is needed (*F* = 1). In the PML model, there is no limit to the number of cells, but the number of strands is limited by the availability of monomers (i.e., nucleotides). The limiting factor is $F=1-S_{tot}/S_{max}$, where $S_{tot}$ is the total number of strands in the whole population and $S_{max}$ is the maximum allowed number of strands. We call this limit global, because it applies equally to all cells in the population. In the PML case, when a cell divides, it is not coupled to the removal of another cell. Instead, all empty cells with *S* = 0 are immediately removed in order to prevent the accumulation of empty cells. In the PCP model, we do not need to immediately remove empty cells because they are eventually removed at random due to the birth and death process of cells.

The PCP and PML models correspond to different assumptions regarding the processes limiting protocell growth. However, we will show below that these two models are surprisingly similar with regard to their error threshold behaviour. Hence, the differences that we observe between the protocells and spatial models do not depend on the process that limits protocell growth.

In the spatial lattice models, there is no division process, but the strands can diffuse from one site to another. The most natural spatial model, which we call SLD (Spatial model with Local Diffusion) has local diffusion of strands between each site and its nearest neighbours that is controlled by a hopping rate *h*. In this model, the parameter *S*_0_ controls the number of strands on any one site. The limiting factor is $F=1-S/S_{0}$, which means that no further replication is possible on a site when *S* *≥* *S*_0_. The local motion of strands leads to a build up of correlations between the contents of one site and its neighbouring sites. This correlation causes the clustering of polymerases, which is part of the reason that the spatial model allows for the survival of polymerases and avoids destruction by parasites. Therefore, it is useful to consider the Spatial Model with Mean Field dynamics (SMF) model as a comparison to this. In mean field dynamics, whenever a strand hops to a different site, it is placed on any other site with equal probability, rather than on a neighbouring site. We have previously studied mean field models with small numbers of strands allowed per site [16,27]. If only one strand is allowed per site, then the mean field model is the same as the well-mixed case, which is not useful, because polymerases are always destroyed by parasites. When up to three strands are permitted per site, the mean field model shows the correct qualitative behaviour, but it is still quantitatively very different from the model with local dynamics. We will show here that when many strands are possible per site (*S*_0_ = 10 or larger in the examples in this paper), there is very little difference between mean field and local dynamics; hence, the mean field approximation is useful. An advantage of the SMF model is that it is possible to give a deterministic solution; whereas, the SLD model requires stochastic simulations.

In both SMF and SLD, the limiting factor on strand growth is locally applied on each site. We also considered a third model, SML (Spatial-Monomer Limited), in which the global supply of monomers limits the strand growth, that is, the limiting factor is $F=1-S_{tot}/S_{max}$, as in the PML model. This corresponds to a case where monomers rapidly diffuse, hence the concentration is the same everywhere. In this way, we can compare protocell and spatial models when the monomer limitation is applied in the same way in the two cases. We did not study a protocell case where there is a local limit on growth, because we are assuming that there is no spatial structure in the protocell population above the level of the cells.

The last column in Table 1 gives the volume, *V*. This is fixed at *V = S_0_* in the spatial models, and it grows in proportion to the number of strands in the PCP and PML models, *V = S*. The volume determines the strand concentrations, and hence the reaction rates, as described in Section 2.2. Although it seems natural to keep *V* constant in the spatial models and to allow it to grow in the protocell models, it is useful for comparison to consider an additional model, PCPCV, in which the volume is kept constant. We will show below that there is a relatively small difference between the PCPCV and PCP models, so the question of whether the protocell volume grows or is fixed is a relatively minor one.

### 2.2. Model Details

In all models, replication requires the encounter of a polymerase with another strand serving as a template, and it produces a strand complementary to the template. Let *p*, *c,* and *x* label the numbers of P, C, and X strands in one site/protocell at a given moment in time, and let $K_{P}\left(p,c,x\right)$, $K_{C}\left(p,c,x\right)$, and $K_{X}\left(p,c,x\right)$. denote the rates of production of P, C, and X strands in this site/protocell. For all models, we may write

$$K_{P}\left(p,c,x\right)=\left(1-M\right)\frac{pcF}{V}k.$$

$$K_{C}\left(p,c,x\right)=\left(1-M\right)\frac{\left(p-1\right)pF}{V}k.$$

$$K_{X}\left(p,c,x\right)=M\frac{pcF}{V}k+M\frac{\left(p-1\right)pF}{V}k+\frac{pxF}{V}k.$$

In the formula for $K_{p}$, *k* is the replication rate per polymerase, *c* is the number of C templates from which new P strands can be produced, and the concentration of polymerases is *p/V*, where *V* is the volume of the cell/lattice site. Note that the rate of increase in the *concentration* of product strands would be proportional to the concentration of the polymerases, *p/V*, times the concentration of the templates, *c/V*. However, *K_p_* is the rate of increase in *number* of strands per cell, not the concentration, so there is an extra factor of *V*. Hence, *K_p_* depends on pc/V, not $pc/V^{2}$. Equivalently, we may say that the rate of increase in the number of product strands is proportional to the concentration of polymerases, *p/V*, times the number of templates, *c*. 

*M* is the probability that a mutation occurs from a P or C to an X during replication. *F* is the limiting factor that prevents the indefinite increase of strands, as discussed in Section 2.1 and Table 1. The formula for $K_{C}$ differs, in that the number of *P* templates is *p*, and the concentration of *other* P strands that can act as polymerases is (*p* − 1)/*V*. The formula for $K_{X}$ includes the term for direct replication of X, plus the terms for creation of X strands by errors in replication of P and C. 

The stochastic simulation of these models proceeds in time steps *δt*. In each time step, births and deaths of strands are separately considered on each site/protocell. The probabilities of adding one P, C, or X strand are $K_{P}\left(p,c,x\right)\delta t$, $K_{C}\left(p,c,x\right)\delta t$, and $K_{X}\left(p,c,x\right)\delta t$. The strands break down at a constant rate, defined as *v* = 1. The probabilities of removal of one P, C, or X strand from a site/protocell are therefore *vpδt, vpδt,* and *vxδt*, respectively. After the birth and death of strands, protocell division occurs in the protocell models and diffusion occurs in the spatial lattice models.

In the protocell models, *S* = *p + c + x* is the current number of strands. Cells with *S* ≥ *S*_0_ undergo random division. Cell division is assumed to be rapid once the split size is reached, i.e., all cells with $S\geq S_{0}$. divide with probability 1 in one time step. The strands from the parent cell are assigned independently with equal probability to one of the two daughter cells. Even though cell division immediately occurs on reaching *S*_0_ strands per cell, it is possible for a small number of cells with $S\geq S_{0}$. to remain in the population after cell division. Firstly, it is occasionally possible to create cells with more than *S*_0_ strands, because replications of P, C, and X strands are independently considered; hence, more than one replication can occur in the same cell in one time step. Secondly, it is possible for the random split to occasionally yield *S*_0_ strands in one daughter and zero in the other; hence, there will sometimes still be *S*_0_ strands after division. 

In the PCP model, we begin with *N* cells, each having one P, one C, and one X. The maximum number of strands that can arise in the PCP model is *NS_0_*. We set *S_max_* in PML to *NS_0_*, where *N* is the fixed population size of the PCP model in order to compare PML with PCP. In the PML model, we begin with $S_{max}/2$ cells, each having one P, one C, and one X.

In the spatial lattice models, the number of lattice sites is analogous to the population size. We consider a square lattice of *N* = *L* *×* *L* sites with periodic boundaries (edges connected in a torus). We begin with one P, one C, and one X in each site. There is a probability *hδt* per time step that a strand diffuses to another site. In the SLD and SML models, strands randomly move to one of the eight sites in their Moore neighbourhood (Figure 1). In the SMF model, strands move to any other site at random. The destruction rate of strands is *v* = 1 in the spatial models, in the same as for the protocell models.

All of these models can be simulated by stochastic methods with finite population sizes and finite numbers of strands. However, in some cases, we can also consider deterministic versions of these models by solving the master equations for the probability distribution *P (p, c, x)* that a site/protocell has *p, c,* and *x* strands of types P, C, and X. This is done in the Appendix A for the protocell model with constant population size and the lattice model with long-distance diffusion.

## 3. Results and Discussion

### 3.1. Error Threshold Behaviour

**Figure 2:**
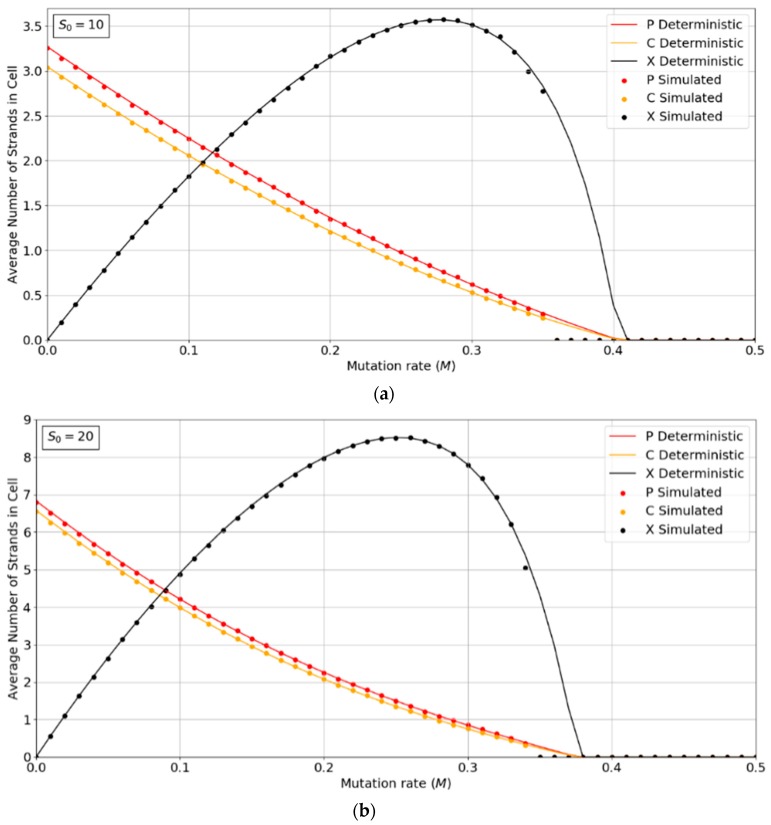
Average numbers of strands per cell in the PCP model. (**a**) *S_0_ =* 10, (**b**) *S_0_ =* 20. k = 25 in both cases. Points are from finite population simulations. Smooth lines are from deterministic theory.

Figure 2a,b show the concentrations of P, C, and X strands as a function of mutation rate, *M*, for the PCP model with *S*_0_ = 10 and 20. The smooth lines are obtained from the deterministic theory in the Appendix A, which applies for infinite populations. The points are measured by simulations with *N* = 1024. These show typical error-threshold behavior. The numbers of P and C strands per cell decrease steadily as the mutation rate is increased, while the number of X strands passes through a maximum. There are always slightly more P than C strands, because of the (*p* − 1) factor in $K_{c}\left(p,c,x\right)$, (i.e., a P cannot replicate itself, whereas a P can replicate all C’s). All three strands die out at the error threshold, *M = M_max_*. The deterministic theory predicts that the strand numbers smoothly decrease to zero as *M* approaches *M_max_*. Close to this point, the expected number of viable cells in a finite population is very small; hence, the finite population simulations are vulnerable to stochastic fluctuations causing the death of the system. The average number of strands in the simulations in the long-time limit is then zero. This causes the simulated systems to die out at slightly smaller values of *M* than is predicted by deterministic models.

**Figure 3:**
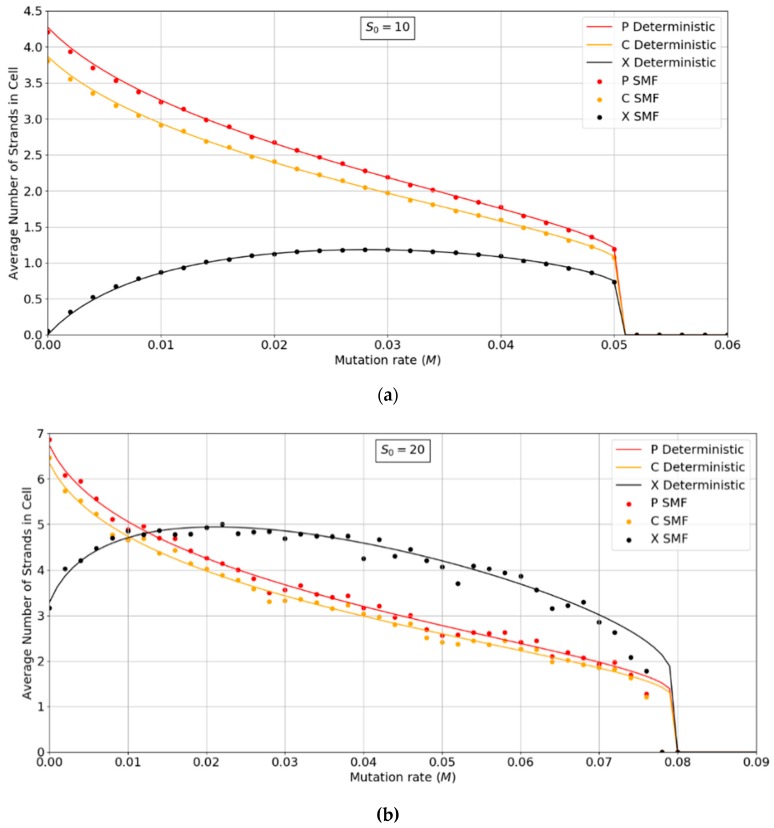
Average numbers of strands per site in the SMF model. (**a**) *N =* 100, *k* = 25, *h* = 0.4 and (**b**) *N* = 400, *k* = 20, *h* = 0.4. Points are from finite population simulations. Smooth lines are from deterministic theory.

Figure 3a,b show the error threshold behavior for the SMF model. In this case, the deterministic theory and the finite population simulation both show a discontinuous transition at the error threshold, i.e., the jump in the curve is not due to stochastic extinction in small populations, as it is in Figure 2. A comparison of Figure 2 and Figure 3 shows that the error threshold is much larger in the protocell model than the lattice model, as we discuss in more detail in Section 3.2. Additionally, of note is the fact that in both the deterministic and mean field versions of the spatial model, at higher S_0_ values, there is a non-zero parasite population present, even at zero mutation rates. In other words there is a coexistence of non-functional parasites with polymerases, even when the parasites are not replenished by mutations from the polymerases. This is a significant difference from the protocell models that were considered in Figure 2, where the parasites are always purged from the systems at zero mutation rates.

### 3.2. Comparison of Error Thresholds in Different Models

**Figure 4:**
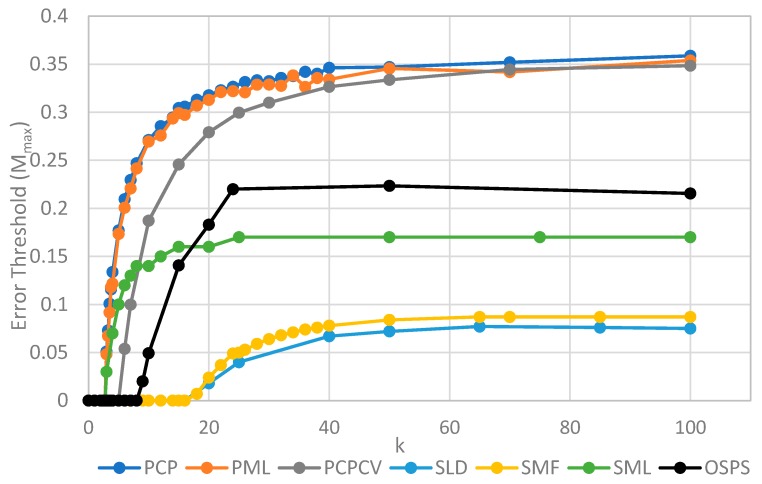
Comparison of the error threshold of the various models studied as a function of the polymerization rate k. S_0_ = 10 in all models except the one per site model, and *h* = 0.4 in the lattice models. All results are from stochastic simulations except for SMF, which results are from the deterministic method. One strand per site (OSPS) is the one strand per site model from [18]. Other models are defined in Table 1.

**Figure 5:**
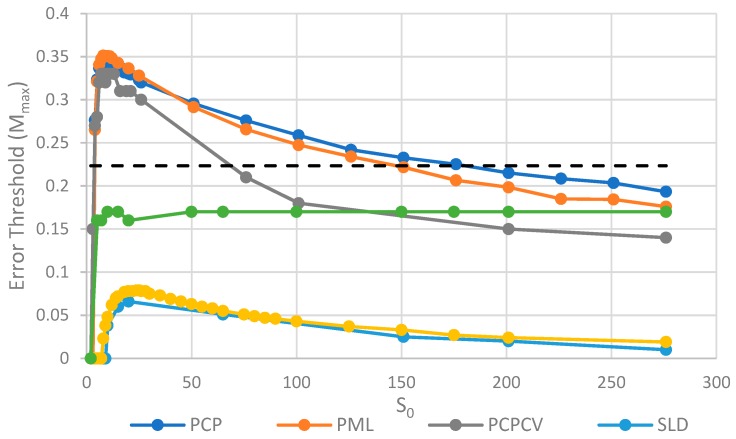
Comparison of the error threshold of the various models studied as a function of *S*_0_. *k* = 25 in all models, and *h* = 0.4 in the lattice models. Results for SMF are obtained from the deterministic method, except for the points with *S*_0_ > 150, where the deterministic method becomes much slower than the stochastic simulation. The results for the other models are obtained from stochastic simulations. OSPS is the one strand per site model from [18]. Other models are defined in Table 1.

The two key properties that we wish to compare between all of the models are the error threshold value, *M_max_* (i.e., the maximum sustainable error probability per replication of the whole sequence) and the minimum catalytic rate, *k_min_*_,_ required for survival of the polymerases. Figure 4 shows *M_max_* measured from simulations as a function of *k*. The estimates of *M_max_* were obtained by running a series of simulations at each value of *k* and then gradually adjusting the mutation rate to zero in on the error threshold. A similar method was used to produce Figure 5, where *S*_0_ was held fixed. 

We will initially discuss the two principal protocell models, PCP and PML, in comparison to the two principal spatial models, SLD and SMF. The other models will be discussed later, because we consider them to be less realistic. The PCP and PML models show a higher error threshold than the SLD and SMF over the whole range of *k* studied, and require the lowest values of *k* to survive. The protocell models are thus “better” for the RNA World, in the sense that survival of the polymerases is substantially easier in the protocells than the lattice models. 

It should be remembered that, even when there are no replication errors (*M = 0*), a minimum value of *k* is necessary for survival, because replication must be faster than the breakdown rate of the strands (*v =* 1). Thus *k_min_* is the value of *k* at which *M_max_* becomes zero. For the PCP and PML models, *k_min_* is approximately 3, whereas it is approximately 18 for SLD and SMF. Thus, there is a substantial range 3 ≤ *k* ≤ 18 where replication is possible in protocells and not in spatial models. All of these rates should be thought of as relative to the breakdown rate, because we have set *v* = 1.

For well-adapted ribozymes, where *k* *≥* *k_min_*_,_ we find *M_max_* is around 0.36 for PCP, but only approx 0.075 for SLD and 0.09 for SMF. Thus, the protocell models are four- to five-fold more tolerant of error. These Figures are per-sequence. If they are converted to per-base error rates, this implies that there is a four- to five-fold greater limit in the maximum length of replicating sequences that can be maintained in protocells relative to spatial models.

Figure 4 also shows the PCPCV model. This model has the volume fixed to *S*_0_ in the same way as it is in the spatial models, and it therefore eliminates a minor difference in the definitions of protocell and spatial models. The error threshold of PCPCV is reduced slightly relative to PCP, but it is still much higher than the spatial models. Therefore, the issue of whether the protocell volume is fixed or grows with the number of strands is only a minor effect. PCP seems more realistic, because in reality a cell cannot keep constant volume when it divides. 

We now turn to the SML model. This has *M_max_* intermediate between the protocell models and the other spatial models, and has *k_min_* that is almost equal to the protocell models. This comparison is interesting from a theoretical point of view, as it highlights the fact that the local limitation on growth that applies in the SLD and SMF models leads to much lower error thresholds than the global limitation in the SML model. However, there are problems with the SML model that mean that is not a biologically realistic model. Replication is fastest on sites with the largest number of polymerases. Strands tend to pile up with very large numbers of strands on a very small number of sites, and with many other sites being empty, as there is no local limit on the number of strands per site in the SML model. This cannot be realistic, because sooner or later, local limits must take effect. Either the monomer limit becomes local, because the concentration of available monomers becomes depleted on sites when there is a lot of replication, or the local limit of space takes effect. Thus, we consider the SLD to be the most realistic of the spatial models, and the comparison between the SLD and the two protocell models as the most valid comparison of the differences between spatial models and protocells.

The final model on Figure 4 is the surface model with only one strand per site (OSPS), taken from Figure 3 of Tupper et al. [18]. In that model, a P strand replicates a strand on a neighbouring site (because there is no other strand on the same site). This model is also intermediate between the protocell models and the SLD model, but again, this seems less realistic than SLD, and it cannot be easily compared with the protocell models, because there is no way of having a protocell with only one strand per compartment.

The parameter *S*_0_, which controls the number of strands per site/cell, has important effects on the error threshold, as shown in Figure 5. For a site/cell to be viable, there must be a minimum of either two P’s or one P and one C. When *S*_0_ is small, there are many sites that are not viable, and the whole system dies out. Once *S*_0_ is above this minimum size for viability, *M_max_* rapidly increases with *S_o_* and then decreases slowly as *S*_0_ becomes large. For very large *S*_0_, each site is a well-mixed model, and there is no more clustering or group selection. Therefore, *M_max_* must tend to zero for very large *S*_0_. The SML model is an outlier here, in that it is not affected by high S_0_ values. In the SML model, the only effect of S_0_ is to determine the total number of strands, because *S_max_* = *S_o_N*, and it does not limit the number of strands on one site, as it does in the other models. The one strand per site model from Tupper et al. [18] is also shown as a comparison, but there is no equivalent of *S*_0_ in this case. It should be remembered that polymerases replicate templates on the neighbouring sites in ref. [18], but on the same site in this paper. Hence, it is not possible to have *S*_0_ = 1 in the spatial models in this paper. Once again, in Figure 5, we see that the PCP and PML models are very similar, and that the PCPCV is only slightly lower than the PCP model. The most useful comparison is between the PCP/PML models and the SLD model, and this shows a substantially larger error threshold for the protocell models, by a factor of 4 to 10.

An interesting observation in Figure 4 and Figure 5 is that the PCP and PML models have almost equal error thresholds, even though the models differ in important respects. For example, all of the cells die with equal probability in the PCP model, but only empty cells die in the PML model. The average replication rate of strands is substantially faster than *v* in the PCP model, because replication has to balance the removal of strands occuring when cells die as well as when individual strands are removed. In contrast, the average replication rate of strands in the PML case is equal to *v,* because the limiting factor reduces this rate to balance the removal of individual strands. Nevertheless, we observe that these apparently large differences do not have a large effect on the error threshold. This is probably because the differences between the models disappear as the mutation rate approaches the error threshold. In the PCP model, the fraction of viable cells becomes very small when $M\to M_{max}$; therefore, the cell division rate is very low, and the rate of removal of strands due to cell death becomes small relative to the rate of removal of individual strands. In the PML model, there is a limiting factor $F=1-S_{tot}/S_{max}$ that is not present in the PCP model. However, when $M\to M_{max}$, the total number of cells is very small and the total number of strands is much less than $S_{max}$. This means that F→1, so this difference between the models also disappears close to the error threshold. 

### 3.3. Effect of Diffusion Rate in the Spatial Models

It can also be seen in Figure 4 and Figure 5 that the SLD and SMF models give quite similar results. This means that the mean field approximation is quite a good one. With the parameters that are chosen in Figure 4 and Figure 5, the error threshold is slightly lower with local diffusion than in the mean field case. However, this depends on the hopping rate *h*, which we have not yet considered. All of the above results were performed with a single value, *h* = 0.4, which was chosen at the beginning of this study, because it gave fairly good survival of polymerases in the spatial models. If *h* is too large, the spatial model becomes well-mixed, and polymerases do not survive. If *h* is too small, there is no spread of strands between sites, and polymerases become extinct independently on each site. The effect of diffusion has also been studied by Branciamore et al. [13] in the case of metabloic replicators, rather than polymerases.

**Figure 6:**
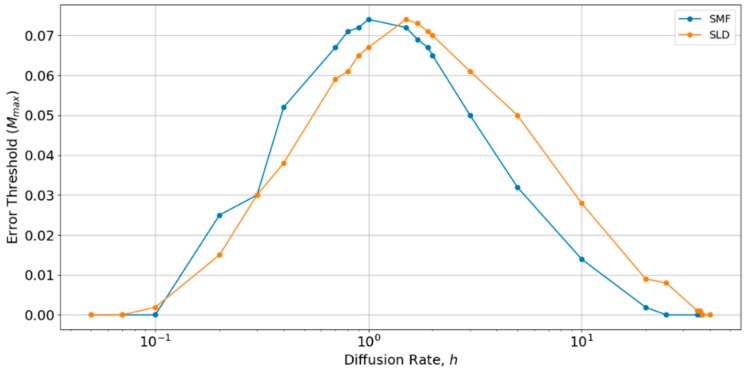
Comparison of the error thresholds of the spatial models with long distance diffusion and local diffusion as a function of the diffusion rate h. Made using S_0_ = 10, k = 25.

Therefore, we performed a further comparison of SLD and SMF models as a function of *h*, as shown in Figure 6. There is an optimum value of *h* close to 1, at which the error threshold is the highest. The optimum *h* is slightly higher for the local diffusion model, but is of order *h ~* 1 in both cases. Below the optimum *h*, the SMF has a slightly higher error threshold (as in Figure 4 and Figure 5), and above the optimum *h*, the SLD model has a slightly higher error threshold, but the difference is always small. The value *h* = 0.4 chosen initially is slightly below the optimum value for both models. Hence, if *h* were tuned to the optimum, the results for SLD and SMF in Figure 4 and Figure 5 would be slightly higher. Nevertheless, the error threshold in the two spatial models is only 0.07 at the optimum *h*, which is still very much less than the protocell models (*M_max_* is approximately 0.32 for the protocell models with *S*_0_ = 10 and *k* = 25, as shown in Figure 2a). Furthermore, there is no reason in nature why diffusion should be tuned to the optimum value. For most of the range of *h*, the error threshold for the spatial models would be even lower than those that are shown in Figure 4 and Figure 5, and the system cannot survive at all (*M_max_* = 0) if *h* is too high or too low. The problem of tuning diffusion does not arise in the protocell models, which is another advantage of protocells.

The fact that the difference between local diffusion and mean field cases is small means that the effect of the dimension of space is small. Our spatial models are all studied on a two-dimensional square lattice. However, we have argued that the spatial model is more usefully thought of as representing a confined geometry, such as pores in a rock rather than molecules stuck on a two-dimensional surface. There is no reason why the lattice needs to be two-dimensional. If we considered a three dimensional lattice, the results would be between the two-dimensional (2D) and mean field cases, i.e., there would be little difference. However, it should be remembered that the spatial clustering mechanism only works if diffusion is very slow. Therefore, it would not apply to an open solution in three-dimensions (3D), in which diffusion and mixing would be rapid when compared to replication.

### 3.4. Rapidly-Replicating Parasites

In all of the previous results, we have assumed that the parasite templates are replicated at the same rate as the functional strands. It seems likely that a substantial fraction of point mutations in the polymerase would disrupt the structure and prevent its function as a ribozyme, but have almost no effect on the ability of the sequence to be a template. However, it is also possible that some mutant sequences would be better templates than the original polymerase. Therefore, in this section, we consider the case where the replication rate for parasites is 2*k*, whereas it remains at *k* for the polymerase and complement. The parasite is thus favored by both mutation and speed of replication. Nevertheless, group selection and clustering effects mean that the polymerase can survive the presence of rapidly multiplying parasites for some parameter values.

**Figure 7:**
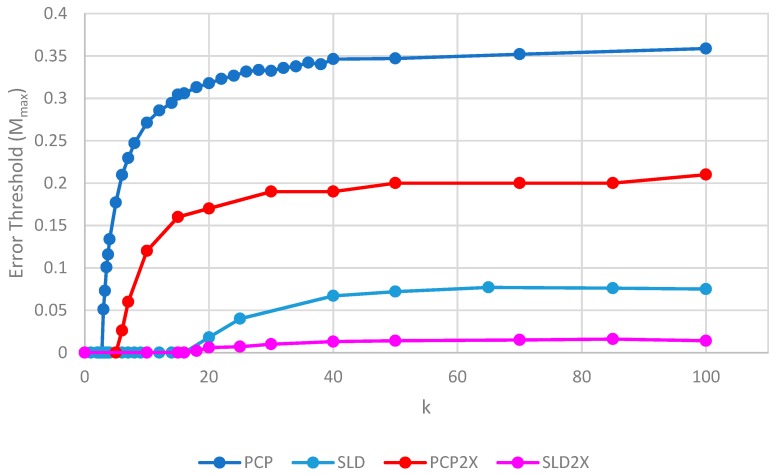
Error thresholds versus *k* for PCP and SLD models in which parasites and polymerases have equal replication rates (same as Figure 4) compared with equivalent models where parasites have double the replication rate of polymerases (denoted PCP2X and SLD2X). Both error thresholds are reduced when the parasites multiply faster, but the SLD model is reduced more, meaning that the relative advantage of the protocells over the spatial model is increased. S_0_ = 10 and *h* = 0.4.

Figure 7 shows the error thresholds of PCP and SLD models as a function of *k* in the case where parasites have the same replication rate as polymerases, together with the equivalent models where the parasites have double the replication rate of polymerases (denoted PCP2X and SLD2X). Both error thresholds are reduced when the parasites multiply faster, but the SLD model is reduced more. For well-adapted ribozymes with high *k*, the ratio of error thresholds for PCP and SLD is 0.36/0.075 = 4.8, whereas the ratio for PCP2X and SLD2X is 0.2/0.014 = 14.3. Thus, the addition of faster replicating parasites increases the advantage of protocells over spatial models.

**Figure 8:**
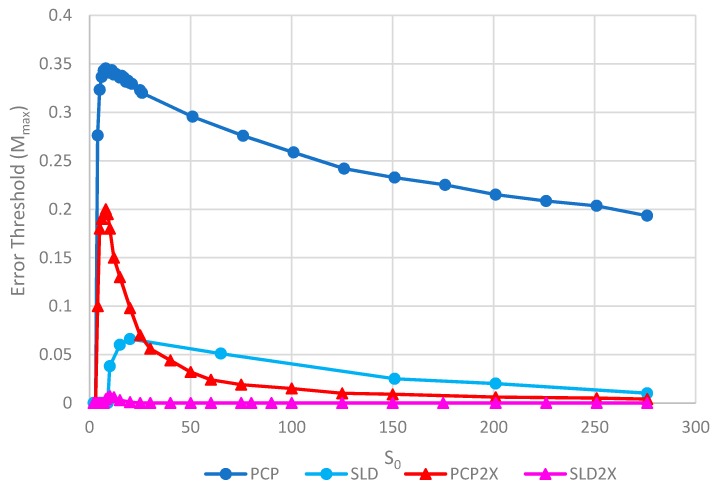
Error thresholds versus *S*_0_ for PCP and SLD models in which parasites and polymerases have equal replication rates (same as Figure 5) compared with equivalent models where parasites have double the replication rate of polymerases (denoted PCP2X and SLD2X). Both error thresholds are reduced when the parasites multiply faster, but the SLD model is reduced more, meaning that the relative advantage of the protocells over the spatial model is increased. Note that *M_max_* = 0 for *S_0_ > 20* for SLD2X, because faster parasites kill the polymerases in the spatial model. k = 25 and *h* = 0.4.

Figure 8 shows the error thresholds for the same models as a function of *S*_0_. The error threshold for PCP2X is substantially reduced relative to PCP for larger *S*_0_. Nevertheless, there is a non-zero error threshold in PCP2X up to at least *S*_0_ = 275. On the other hand, there is only a very narrow range of *S*_0_ (approximately 7–20) where the error threshold is non-zero for SLD2X, and even within this range, the error threshold is extremely low. For *S_0_ >* 20 in the SLD2X model, fast replicating parasites multiply and lead to destruction of the polymerases (and themselves), even in the limit of zero mutation rate. We allowed the system to reach a steady state with only P and C strands present to test the limit of zero mutation rate. A very small number of parasites were then added, and replication continued with zero mutation rate. For *S_0_ >* 20, the initial few parasites multiply and destroy the system, even though there is no further production of parasites by mutation. In contrast, there is a finite error threshold for the PCP2X model at high *S*_0_, as we just noted. Thus, once again, the advantage of the protocell over the spatial model is increased when we consider faster replicating parasites.

## 4. Conclusions

Although the mechanisms by which compartments and spatial clustering promote the survival of polymerases (or other kinds of cooperative replicators) have been understood for some time, there has not been much quantitative comparison of the two. It becomes apparent that it is necessary to clearly specify which factors limit the growth of strands when designing models to allow for this quantitative comparison. The limiting resource could simply be space, as is likely to be the case if there is a maximum number of strands that can fit in the space represented by one lattice site. Alternatively, the limit may be the availability of monomers to synthesize the strands, or it may be the availability of another molecular resource, such as lipids, which limits the growth of protocells before the supply of monomers runs out. 

It is also important to consider whether the limiting factor acts globally on the whole population, or locally on one protocell/site at a time. In the spatial models, it seems natural to apply the limitation locally, as in the SLD and SMF models, however we also considered the SML case where a global monomer limitation was applied. The survival of polymerases was somewhat easier when the limit was global, but this model does not seem realistic. The spatial models are intended to represent restricted geometries where diffusion will be slow, such as crevices in rocks. A lattice site would represent a region in which strands can interact with one another. Space is obviously limited in such environments. The diffusion of strands has to be slow in spatial models, otherwise the system becomes well-mixed and polymerases do not survive. Although the diffusion of monomers might be faster than that of strands, it is still finite. Thus the monomer limitation in spatial models has to be local. The locally-limited spatial models have a much lower error threshold and a much higher minimum catalytic rate that the protocell models. Hence, our principle conclusion that polymerase survival is much easier in protocells than in spatial models with restricted geometry, such as pores in a rock.

In protocell models, it seems natural to assume that the limiting factor is global, as will be the case if the protocells are free to move in the surrounding medium, and if the supply of either lipids or monomers to the protocells is rapid and well-mixed. There is no restrictive geometry or surface binding to slow things down in this case. A somewhat surprising finding of this paper is that the PML model, where strand growth is limited by monomers, and the PCP model, where protocell growth is limited by factors other than monomer supply, give such similar results in terms of the error threshold. Hence, our conclusion is that the advantages of protocells that were found in this paper are robust to model variations.

Although both protocell models and spatial models have been widely studied, there has been little previous quantitative comparison. This paper enables us to make that comparison in a novel way and to distinguish carefully the different factors that limit replication. We will emphasize several detailed aspects of the models that emerge from this comparison. There are two different kinds of transitions that occur at the error threshold: either the strand concentrations go continuously to zero (as in Figure 2) or there is a discontinuous jump (as in Figure 3). Figure 3b also shows the unexpected result that parasites can coexist with polymerases in the limit of zero mutation rate where they are no longer being created. This occurs in the spatial models with large enough *S*_0_ but not in protocell models. In the case where parasites multiply faster than polymerases, the advantage of the protocells over the spatial models is increased (as in Figure 7 and Figure 8). In Figure 8, there is a qualitative difference between the protocells (PCP2X) and spatial model (SLD2X). For the protocells, there is a finite error threshold, even for the largest *S*_0_ considered, whereas parasites destroy the spatial system, even in the limit of zero mutation rate.

The results in this paper are somewhat different to those that were obtained from the Cellular Potts Model (CPM) [14], because they consider different cases. The CPM begins with a spatial model of only one strand per site, and adds cellular compartments on top of this. It is found [14] that these surface models with and without compartments do not qualitatively differ from one another in respect to their stability against mutations. This is best thought of as representing a case where strands are stuck to surfaces. In contrast, we have referred to our lattice models as ‘spatial’ not ‘surface’, because they model a case where spatial clustering occurs due to slow diffusion in restricted geometries. In our case, mixing is fast locally, but slow on a global scale. Fast mixing on the local scale is equivalent to fast mixing inside a single protocell. This makes the protocell and spatial models equivalent on the local scale and allows for direct comparison. Our models clearly separate the effects of diffusion between sites from the effects of group selection and cell division, and show that group selection is noticeably better as a means of limiting the growth of parasites.

The diversity and success of cellular life on Earth is evident and there is no evidence for distributed living systems on surfaces. This paper goes some way to showing why this is. It also fits with our previous study [17] of interacting polymerase and nucleotide synthetase ribozymes, where we pointed out that the survival of two complementary types of unlinked ribozymes is possible on a surface, but is difficult, because it requires joint spatial patterns to form. There is no equivalent problem if the ribozymes are in compartments. Hence, we expect evolution from single replicators to genetic systems involving multiple types of ribozymes to be easier in protocells.

We are very pleased to have the opportunity to contribute to this volume dedicated to Prof. David Deamer. The Origins Institute at McMaster has benefitted greatly from David’s help and advice over many years, and we are very grateful for his scientific input, friendship, and enthusiasm. David has made many important contributions to the understanding of the origins of life. In particular, he has long been an advocate of the importance of membranes to early life [37,38,39]. His recent work has shown that lipid membranes can provide an environment in which RNA polymerization becomes possible [40]. The stability of membranes in fresh water conditions and the possibility of wetting and drying cycles occurring in shallow water has led to his current view that life began in freshwater pools associated with volcanic islands [41,42]. Under these conditions, it is likely that membranes were available to encapsulate the earliest replicating polymers at the time of the origin of life. This ties in with the conclusions of the current paper, in which we have shown that encapsulation greatly increases the ability of RNA replicators to survive when the replication accuracy is low. Thus, we conclude that the presence of protocells is beneficial to the development of early life, and it seems quite likely that the first replicating polymers may have functioned inside lipid vesicles from the outset.

## Figures and Tables

**Table 1:** Overview of models in this study.

Model	Dynamics	Limiting Factor	Volume
PCP—Protocells with Constant Population	Division when $S\geq S_{0}$ *N* fixed	No limit, F=1	Grows with cell V=S
PCPCV—Protocells with Constant Population and Constant Volume	Division when $S\geq S_{0}$ *N* fixed	No limit, F=1	Constant $V=S_{0}$
PML—Protocells Monomer Limited	Division when $S\geq S_{0}$ *N* variable	Global limit, $F=1-S_{tot}/S_{max}$	Grows with cell V=S
SLD—Spatial Model with Local Diffusion	Local diffusion rate *h*	Local limit, $F=1-S/S_{0}$	Constant $V=S_{0}$
SMF—Spatial Model with Mean Field dynamics	Mean field diffusion rate *h*	Local limit, $F=1-S/S_{0}$	Constant $V=S_{0}$
SML—Spatial Model Monomer Limited	Local diffusion rate *h*	Global limit, $F=1-S_{tot}/S_{max}$	Constant $V=S_{0}$

## Appendix A

In the limit where the number of lattice sites or protocells becomes infinite, it is possible to calculate probability distribution P(p,c,x) that a site/protocell will have *p*, *c* and *x* molecules of types P, C and X. For the protocell model with constant population, we may write the rate of change of the probabilities due to birth and death of strands as

$$\frac{dP\left(p,c,x\right)}{dt}=K_{P}\left(p-1,c,x\right)P\left(p-1,c,x\right)+K_{C}\left(p,c-1,x\right)P\left(p,c-1,x\right)+K_{X}\left(p,c,x-1\right)P\left(p,c,x-1\right)$$

$$-\left(K_{P}\left(p,c,x\right)+K_{C}\left(p,c,x\right)+K_{X}\left(p,c,x\right)\right)P\left(p,c,x\right)$$

+v(p+1)P(p+1,c,x)+v(p+1)P(p,c+1,x)+v(x+1)P(p,c,x+1)−v(p+c+x)P(p,c,x).

The rate of production of cells of type (p,c,x) by random division of cells of type $\left(p_{0},c_{0},x_{0}\right)$ is

$$b\left(p,c,x|p_{0},c_{0},x_{0}\right)=2\frac{p_{0}!}{2^{p_{0}}p!\left(p_{0}-p\right)!}\frac{c_{0}!}{2^{c_{0}}c!\left(c_{0}-c\right)!}\frac{x_{0}!}{2^{x_{0}}x!\left(x_{0}-x\right)!}.$$

All cells with at least $S_{0}$ strands divide with probability 1 in one time step. The total rate of production of cells of type (p,c,x) by division of all cells with at least $S_{0}$ strands is

$$B\left(p,c,x\right)=\sum_{p_{0}\geq p;c_{0}\geq c;x_{0}\geq x;p_{0}+c_{0}+x_{0}\geq S_{0}}b\left(p,c,x|p_{0},c_{0},x_{0}\right)P\left(p_{0},c_{0},x_{0}\right).$$

As the population is fixed, the total rate of removal of cells when other cells divide is equal to the total division rate:

$$B_{tot}=\sum_{p_{0}+c_{0}+x_{0}\geq S_{0}}P\left(p_{0},c_{0},x_{0}\right)$$

The change in probability in one time step due to cell division is therefore $\Delta P\left(p,c,x\right)=B\left(p,c,x\right)-B_{tot}P(p,c,x)$, for cells with $p+c+x<S_{0}$, and $\Delta P(p,c,x)=B\left(p,c,x\right)-B_{tot}P(p,c,x)-P(p,c,x)$, for cells with $p+c+x\geq S_{0}$. Thus the probability of each cell type after one time step, accounting for both strand birth and death and cell division is

$${P\left(p,c,x\right)|}_{t+\delta t}={P\left(p,c,x\right)|}_{t}+\delta t\frac{dP\left(p,c,x\right)}{dt}+\Delta P\left(p,c,x\right).$$

To solve the model by this deterministic method, we iterate forward in time till the stationary state is reached.

The spatial lattice model can also be solved deterministically if we make a mean field assumption, i.e., there is a single central site surrounded by a homogenous environment. The terms for birth and death of strands are the same as for the protocell case. Terms for diffusion into and out of the central site are added, as follows.

$$\frac{dP\left(p,c,x\right)}{dt}=birth\;and\;death\;terms$$

+h(p+1)P(p+1,c,x)+h(p+1)P(p,c+1,x)+h(x+1)P(p,c,x+1)−h(p+c+x)P(p,c,x)

$$+h\bar{p}P\left(p-1,c,x\right)+h\bar{c}P\left(p,c-1,x\right)+h\bar{x}P\left(p,c,x-1\right)-h\left(\bar{p}+\bar{c}+\bar{x}\right)P\left(p,c,x\right)$$

Here, $\bar{p}$, $\bar{c}$ and $\bar{x}$ are the current mean values of the numbers of strands per site across the whole lattice: $\bar{p}=\sum_{p,c,x}pP(p,c,x)$, $\bar{c}=\sum_{p,c,x}cP(p,c,x)$ , and $\bar{x}=\sum_{p,c,x}xP(p,c,x)$. The mean field method has also been used in similar models by McCaskill et al. [43] and in our previous work [16,27].
